# Optimization and Performance Evaluation of Melt-Blown Fiber Membranes for Leukocyte Separation from Whole Blood

**DOI:** 10.1021/acsomega.5c13313

**Published:** 2026-05-13

**Authors:** Firdevs Mert Sivri, Numan Hoda, Mehmet Ali Selek, Fatma Kara

**Affiliations:** † Faculty of Pharmacy, Department of Basic Pharmaceutical Sciences, 52994 Suleyman Demirel University, 3200 Isparta, Türkiye; ‡ Faculty of Engineering, Department of Materials Science and Engineering, 37502 Akdeniz University, 07058 Antalya, Türkiye; § AntıpTechnological and Medical Goods Co., Antalya, Türkiye

## Abstract

Leukocytes need to be eliminated from blood components in order to lower the risk of adverse transfusion responses and guarantee patient safety during transfusion. Nowadays, nonwoven fabric filters are typically used to remove leukocytes from whole blood and blood components. Research on these types of filters, filter materials, and filtration mechanisms is increasing day by day to further enhance leukocyte removal. In this work, melt-blown nonwoven layers were produced from polybutylene terephthalate (PBT) and polyamide-12 (PA-12) and combined into eight leukocyte filter prototypes with varying architectures, such as PBT-only stacks and PBT/PA-12 multilayer combinations with ∼60–100 total layers and average pore diameters in individual layers of ∼14.7–67.8 μm. The melt-blown processing conditions (air pressure, polymer flow rate, die–collector distance, and collector speed) were screened for their influence on fiber diameter and layer thickness to produce filter-grade nonwovens. Filter morphology, pore size distribution, thermal stability, and surface charge (pHpzc) were analyzed, and filtration performance was assessed using gravity-driven whole-blood processing (450 mL per unit). Filters with both PBT and PA-12 layers removed >99% leukocytes with <10% erythrocyte loss, which is superior to PBT-only filters. This enhanced performance is ascribed to the synergistic surface properties of the mildly basic PBT and the nitrogen-containing PA-12, which facilitate adsorption-mediated leukocyte capture in the multilayer filter. In summary, these findings show that melt-blown PBT/PA-12 multilayer filters offer a scalable platform for efficient leukoreduction with low erythrocyte loss.

## Graphical abstract

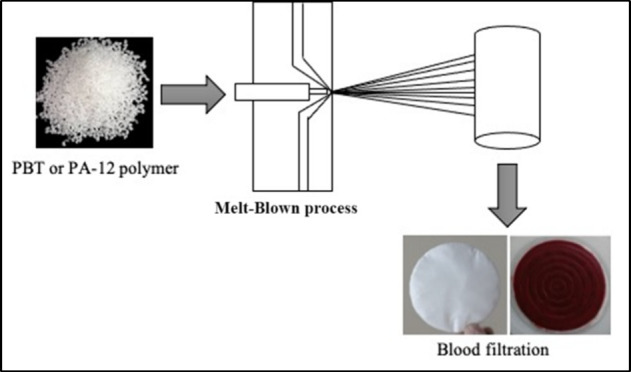

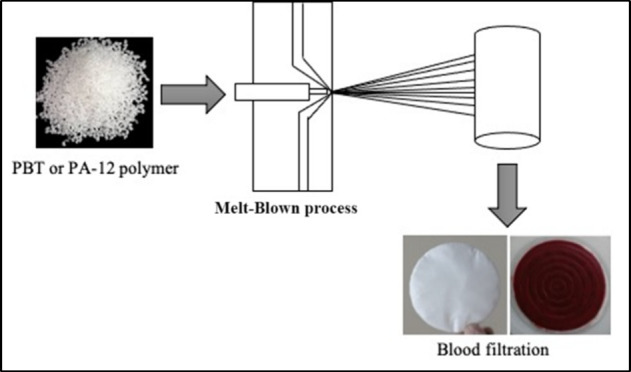

## 1 Introduction

Blood transfusions are defined as the administration of whole blood or specific blood components from a donor to a patient who requires blood due to conditions such as anemia, hemophilia, trauma, or surgical procedures. In the early days of transfusion practice, whole blood was often directly administered to patients instead of the specific blood components they needed.^1,2^ However, in modern medical practice, particularly in developed countries, transfusions typically contain only essential blood components such as red blood cells, platelets, or plasma. Despite separation of blood components, these products contain residual leukocytes. Studies have shown that residual leukocytes present in transfused red blood cell and platelet units can cause adverse effects in recipients.^3,4^ These effects include the possibility of causing febrile nonhemolytic reactions such as fever and chills, alloimmunization, transfusion-associated graft-versus-host disease, immune modulation, increased risk of infection, and cytokine-mediated inflammatory responses.^3,5,6^

At present, several methods are employed to prepare leukocyte-reduced blood from whole blood, including differential centrifugation, sedimentation, freezing and thawing, cell washing, and filtration.^7–9^ While techniques such as centrifugation and buffy coat removal (approximately 10^8^ WBCs, 1 log reduction), washed red blood cell concentrates (10^7^ WBCs, 1–2 log reduction), and frozen deglycerolized red cells (10^6^–10^7^ WBCs, 2–3 log reduction) achieve varying levels of leukocyte removal, filtration remains the most effective and widely adopted approach, as it meets the current leukocyte depletion standard of fewer than 5 × 10^6^ WBCs with loss of not more than 10% RBCs per unit of blood component in US while it is 1 × 10^6^ WBCs in EU.^10,11^ Therefore, filtration is regarded as the most efficient and reliable technique for achieving clinically acceptable leukoreduction, offering high removal efficiency, preservation of blood component quality, and suitability for large-scale clinical applications.

The primary mechanisms for leukocyte removal in blood filtration are sieving and adhesion. Sieving occurs by physically retaining leukocytes larger than the filter pores, while adhesion is the process of removing leukocytes by binding to the surface of the filter material through electrostatic and physical interactions.^12–15^ In a study by Watanabe et al.,^16^ when leukocytes were filtered from whole blood or red blood cells using a nonwoven polyester fiber filter, the leukocyte retention rate increased as the fiber diameter decreased. Furthermore, the leukocyte retention rate exceeded 95% when the nonwoven fiber diameter was less than 3 μm.

Nonwoven fibers are widely used in leukocyte filtration due to their high filtration performance. The active filtration layers of nonwoven filters used in leukocyte filtration generally consist of microfibers produced by the meltblown method. The melt-blown process is a nonwoven fabric production technique in which thermoplastic polymer filaments are extruded and attenuated using high-velocity hot air. Polymers such as polypropylene, polyethylene, polyethylene terephthalate, polybutylene terephthalate, polystyrene, polyurethane, and polyamide are commonly used to produce nonwoven materials via the melt-blown method.^17^ The diameter of microfibers produced by this technique typically ranges from as small as 0.1 μm to as large as 10–15 μm but most commonly falls within the 2–4 μm range.^18,19^ The ability to control fiber diameter and web density allows for a defined pore distribution, making melt-blown materials highly suitable for filtration applications.^20^

Leukocyte adhesion to fibrous filters is known to be greatly affected by surface chemistry and charge, as well as pore morphology. Surfaces bearing hydroxyl, carbonyl, and amine functional groups have been shown to favor leukocyte adhesion, whereas sulfonated surfaces inhibit adhesion.^21,22^ In addition, basic nitrogen-containing groups may improve selective leukocyte retention over erythrocytes, supporting an adsorption-dominated capture process in depth-type nonwoven filters.^23–25^ In this study, PA-12, which contains nitrogen on its surface, and PBT, which exhibits basic surface properties, were selected for filter production.

The specific benefit of the PBT/PA-12 multilayer approach employed in this study is that it combines the mildly basic surface properties of PBT with the nitrogen-containing amide groups of PA-12 in a layered depth architecture, which can provide chemically heterogeneous high-area contact pathways that may improve adsorption-mediated leukocyte capture while preserving permeability and preventing RBC loss. This can be done in a manner that is still amenable to melt-blown manufacturing and filter fabrication.

The primary objective of this study was to produce a high-performance leukocyte filter composed of fiber layers of both PBT and PA-12 polymers. To achieve this goal, fibers were produced from PBT and PA-12 polymers using a meltblown technique, and leukocyte filters were fabricated from the obtain fiber layers. Furthermore, blood filtration experiments were conducted to evaluate the performance of the produced filters.

## 2 Materials and Methods

### 2.1 Materials

In this study, a 38 cm-wide Biax Melt-Blown Pilot Line System was used, equipped with 720 nozzles (each with an internal diameter of 150 μm) arranged in four rows and integrated with air curtains. PBT supplied by Celanese and PA-12 supplied by Arkema polymers were used for melt-blown fiber production. The PBT polymer showed a melt flow rate of 1200 g/10 min and a relative density of 0.9 at 20 °C, whereas the PA-12 polymer showed a density of 1.3 g/cm^3^ and a melt flow rate of 360 g/10 min. Both polymers possessed suitable rheological and physical properties for the production of fine fibers under high-throughput melt-blown processing conditions. The Red Crescent Blood Center supplied the blood samples utilized in the leukocyte filtering performance tests.

### 2.2 Production of PBT and PA-12 Fibers

Fiber production from PBT and PA-12 polymers was carried out using the melt-blowing technique. Initially, a series of experiments were conducted to determine the optimum processing temperatures for each polymer. Based on these experiments, the optimum fiber production temperatures for PBT and PA-12 were determined, as summarized in Table 1.

**Table 1:** Optimum Operating Temperatures of Polymers in the Melt Blowing

Polymer	Zone 3 (°C)	Zone 2 (°C)	Zone 1 (°C)	Air Temp. (°C)	Die (°C)	Clamp (°C)
PBT	250	245	242	250	250	250
PA-12	220	215	210	220	220	220

The average fiber diameters and layer thicknesses of fibers produced by the melt blowing method are affected by various parameters such as the pressure of the blown air, the polymer flow rate, the distance between the die head and the collector, and the rotation speed of the collector.^26^ Therefore, a series of fiber tensile tests were conducted in melt blowing for both PBT and PA-12 polymers, and each of these parameters was systematically varied.

### 2.3 Characterization of Fibers Produced from PBT and PA-12 Polymers

#### 2.3.1 Fiber Diameter and Layer Thickness Analysis

A Leica DM750P optical microscope was employed to determine fiber diameters. To account for heterogeneity on the web, the nonwoven sample was subdivided into three areas across the web width. Micrographs were taken from several randomly chosen fields of view in each area at the same magnification and imaging conditions. Fiber diameters were determined from the micrographs using image analysis software. A total of 100 individual fiber diameter values were obtained per sample using a balanced sampling design.

Layer thickness was determined using an Olympus BX41 M microscope on cross-sectional images. For each nonwoven layer, samples were obtained from the same three areas on the web and layer thickness was determined at several locations on each cross-section. A total of 100 thickness values were obtained per sample using a balanced sampling design as described above.

#### 2.3.2 pHpzc Analysis

The surface charge properties of the fibers, which are critical for leukocyte adhesion and detachment during blood filtration, were evaluated by measuring their pHpzc. The pH shift method was used to determine the pHpzc of PBT and PA-12 fibers. For this purpose, a series of previously cleaned Erlenmeyer flasks were filled with 20 mL of 0.01 M NaNO_3_ solution. Using diluted HCl and NaOH solutions, the initial pH values of the solutions were adjusted to a range of values (generally between pH 2 and pH 12) by continuously monitoring with a pH meter. After adjusting the pH, 0.05 g of PBT fiber was added to each flask and the solution was shaken in a shaking water bath under vacuum at room temperature for 24 h. After reaching equilibrium, the final pH values of the solutions were measured. The same procedure was applied to the PA-12 fiber. The same procedure was applied for PA-12 fibers to determine pHpzc value.

#### 2.3.3 TGA/DTA Analysis

The thermal stability of PBT and PA-12 fibers was investigated using a NETZSCH STA 449F3 thermal analyzer. For thermogravimetric analysis (TGA) of PBT and PA-12 fibers, each fiber sample was placed in a sample holder and heated from 30 to 300 °C under a nitrogen atmosphere at a heating rate of 10 °C/min to determine their thermal stability. For differential thermal analysis (DTA), the samples were similarly heated from 30 to 400 °C under a nitrogen atmosphere at a heating rate of 10 °C/min.

#### 2.3.4 Porosity Measurement via Mercury Porosimetry

The porosity analysis of the PBT and PA-12 fiber layers used in filtration was performed using a Micromeritics AutoPore IV 9500 mercury porosimeter. In the porosimetry analysis of PBT and PA-12 fibers, the contact angle of mercury was set to 130°, the surface tension of mercury was taken as 485 dyn/cm, and the maximum applied pressure was 60,000 psi.

### 2.4 Preparation of Filters

Filters are prepared by stacking predetermined layers of fibers on top of each other. The experiments were designed considering two main factors: (i) the number of layers forming the filter and the pore size of each layer and (ii) the number of layers from two different polymer fiber types used in the filter. Since the total thickness of the filter influences leukocyte separation efficiency, optimization studies were performed by varying the number of layers to investigate its effect on leukocyte removal capacity. Additionally, to evaluate the impact of layers composed of different polymers on leukocyte separation, filters were assembled by varying both the number of layers from each polymer and their sequential arrangement.

### 2.5 Leukocyte Filtration

The prepared filters were placed into a custom-designed Teflon filtration setup, as illustrated in Figure 1.

**Figure 1:**
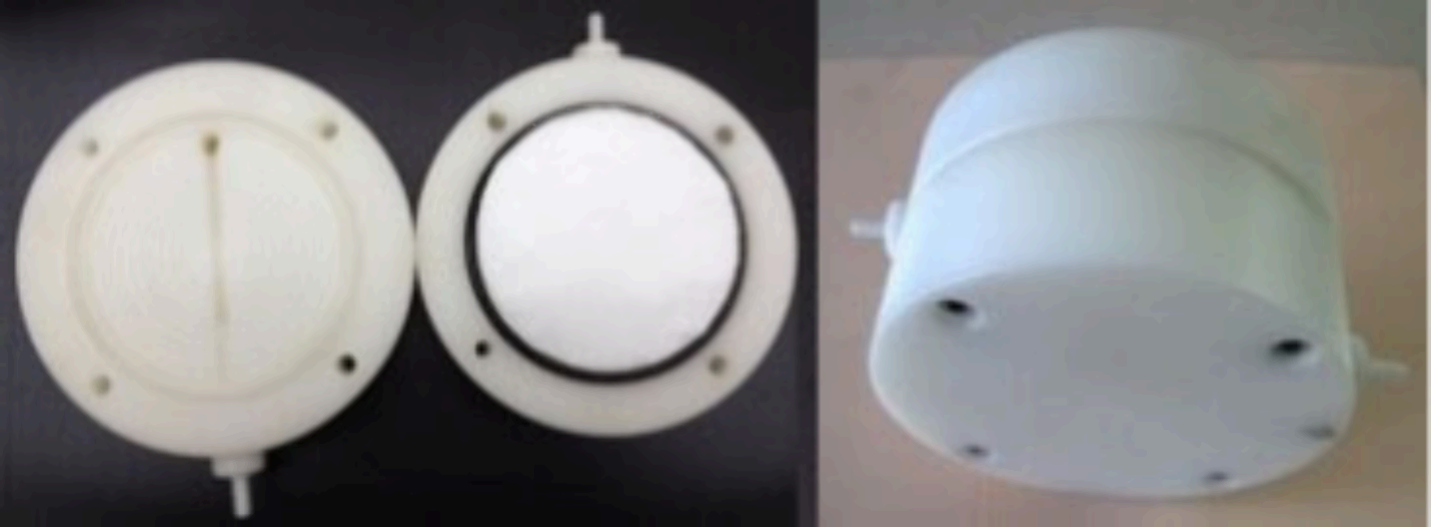
Structure of the fabricated Teflon housing and the placement of the filters inside the housing.

The whole blood (450 mL) was filtered with the developed filter prototypes under gravity-driven flow in a specially designed Teflon housing (Figure 1). The filtration process was carried out at room temperature (24 °C) without any external pumping or pressure other than the hydrostatic pressure from the blood bag. In each filtration experiment, the whole 450 mL was passed through the filter and collected in a sterile blood collection bag. The total filtration time taken was measured and was around 30 min for the whole unit. The appearance of the filter before and after filtration is shown in Figure 2.

**Figure 2:**
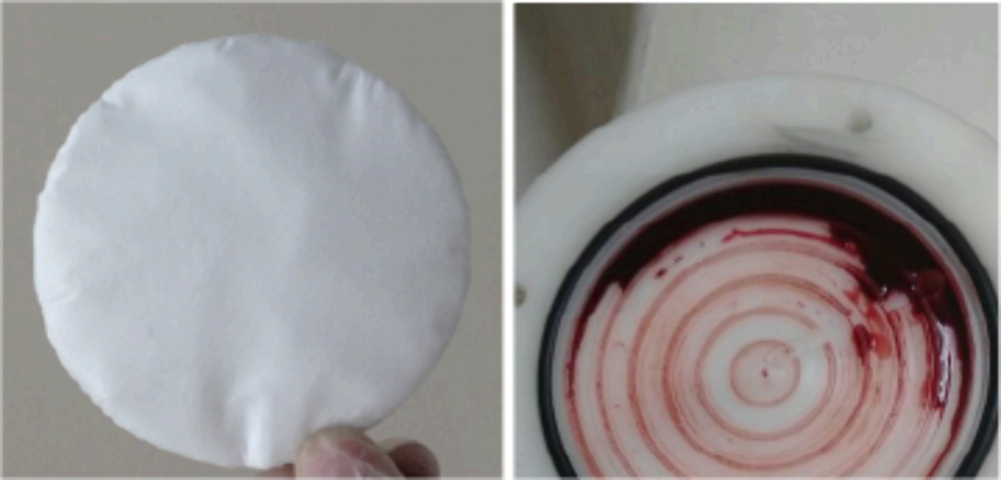
Appearance of the filter before and after filtration.

Hemogram analyses were carried out on the blood samples collected before filtration and from the collected blood after filtration. Leukocyte removal (%) and erythrocyte loss (%) were calculated based on the comparison of the pre- and postfiltration values of the leukocyte and erythrocyte counts for each filtration setup.

## 3 Results and Discussion

Fiber production was achieved by varying parameters such as air pressure, polymer flow rate, die head-collector distance, and collector rotation speed to create fibers with different diameters and layer thicknesses using the meltblown method. To obtain suitable fiber properties for leukocyte filtration of PBT fibers, the effect of each process parameter on fiber diameter in the melt-blown process was investigated. however, a stable and continuous fiber production in PA-12 fibers could only be accomplished within a single process range. The production parameters and characteristics of fibers with suitable diameters and layer thicknesses to fabricate the filter prototype are listed in Table 2.

**Table 2:** Parameters Used in Melt Blowing and Properties of Fibers Obtained with These Parameters^a^

Polymer	Air Pressure (PSI)	Polymer Flow Rate (g/dak/orf.)	Distance (cm)	Collector Rotation Speed (m/min)	Avg. Fiber Diameter (μm)	Avg. Fiber Layer Thickness (μm)
PBT-1 (35)	5	20	35	30	3.1 ± 3.5	26.9 ± 4.4
PBT-2 (42)	5	40	16	30	2.6 ± 1.5	25.5 ± 5.6
PBT-3 (58)	15	30	16	20	1.5 ± 0.8	36.6 ± 3.6
PBT-4 (50)	5	30	16	20	2.0 ± 0.8	29.1 ± 7.0
PA-1	5	20	16	5	5.2 ± 3.3	61.0 ± 5.7

a Mean ± SD is calculated from *n* = 100 individual fiber diameter measurements per sample (no outlier removal).

The pHpzc analysis results revealed the influence of surface characteristics of the polymers used on leukocyte filtration performance. The pHpzc value of PBT fibers was found to be 7.63, indicating that these fibers possess mildly basic surface properties (Figure 3A). In contrast, PA-12 fibers exhibited a pHpzc value of 7.03, suggesting that they possess nearly neutral surface characteristics (Figure 3B). It is known that basic amine-rich surfaces supports strong adhesion interactions with leukocytes compered to neutral surfaces.^25^ Also, as reported in the literature,^27^ fibers containing nitrogen-based functional groups on their surface exhibit selective leukocyte retention over erythrocytes when the nitrogen atom-to-surface ratio (by weight) is between 0.2% and 4.0%. Accordingly, despite the neutral pHpzc, PA-12 has significant potential for selective leukocyte capture due to the presence of nitrogen-containing groups on its surface. When combining these two polymers, the basic nature of PBT contributes to strong electrostatic interactions, while the structural characteristics of PA-12 enhance selective leukocyte adhesion.

**Figure 3:**
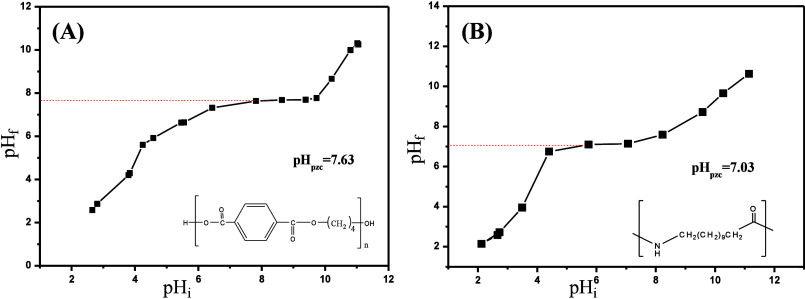
PHpzc graph of (A) PBT and (B) PA fibers produced by the melt-blown technique.

To determine the thermal stability of the PBT and PA-12 fiber layers used in the filtration process, TG/DTA analyses were performed. For this analysis, thermal characterizations were conducted on the PBT and PA-12 samples intended for use in the filter structure. As shown in Figure 4, the results demonstrated that up to 300 °C, neither PBT nor PA-12 fibers significantly degraded.

**Figure 4:**
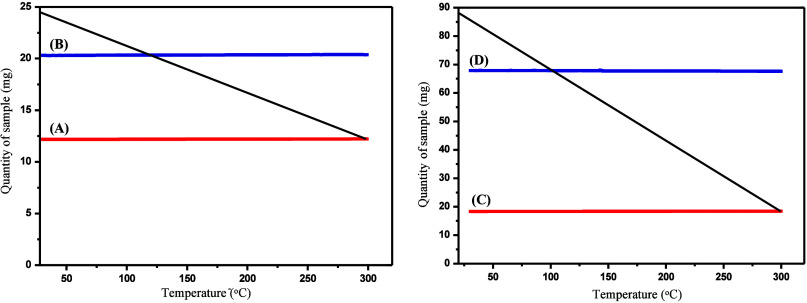
TGA graph of (A) PBT polymer, (B) PBT fiber, (C) PA-12 polymer, and (D) PA-12 fiber.

The DTA technique examines the temperature difference between a sample and a reference material as a function of the applied temperature. DTA is a powerful and commonly used method in polymer studies and characterizations. In the DTA analysis of PBT fibers, the crystallization temperature was observed at 213 °C, and the melting temperature at 231 °C. Additionally, the onset of thermal degradation for PBT fibers was noted to begin at approximately 379 °C (Figure 5A). For PA-12 fibers, the DTA analysis revealed a crystallization temperature of 171 °C and a melting temperature of 190 °C. It was also observed that PA-12 fibers exhibited thermal stability up to 400 °C without significant degradation (Figure 5B).

**Figure 5:**
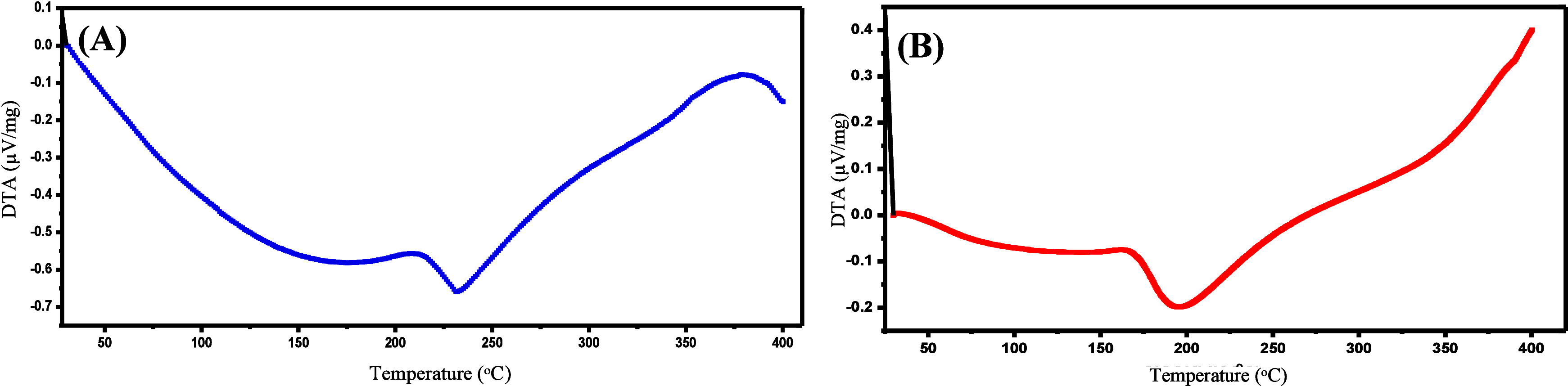
DTA graph of (A) PBT fiber and (B) PA-12 fiber

In leukocyte filtration, the average pore size of the layers that make up the filter plays a significant role in overall filtration performance. As the average pore size decreases, the risk of filter clogging increases. Conversely, as the pore size increases, adhesion forces decrease, which negatively impacts the filtration efficiency. As seen in Table 3, filters with average pore sizes ranging from 14.7 to 67.7 μm were determined and stored for use in blood filtration applications. As seen in Table 3, increasing the number of layers did not result in a decrease in porosity of PA-12 filters. This can be attributed to the larger fiber diameter of the PA-12, which provides a more open structure and prevents a significant decrease in pore size even with increasing the number of layers.

**Table 3:** Porosity Values of Fibers to Be Used in the Filter

Sample	Average Pore Diameter (μm)	Porosity (%)
25 layers of PBT-1	34.1	83.1
50 layers of PBT-1	26.0	76.5
25 layers of PBT-2	28.9	82.0
25 layers of PBT-3	17.8	76.1
50 layers of PBT-3	14.7	72.5
20 layers of PBT-4	21.3	79.9
25 layers of PA-12	53.0	81.8
50 layers of PA-12	67.8	82.9

Filtration experiments using blood samples were conducted to evaluate the performance of filters prepared with different numbers of layers and fiber layers made from two different polymers, and the results are presented in Table 4. The performance evaluation of the produced leukocyte filters was carried out by comparing the leukocyte and erythrocyte counts before and after filtration. Increasing the number of filter layers generally increased leukocyte removal because more layers increased the surface area available for leukocytes to contact through the filter. However, excessive layer thickness created resistance to fluid flow, creating mechanical difficulties for erythrocytes to pass through.

**Table 4:** Blood Filtration Results of the Produced Leukocyte Filters

		Before Filtration				After Filtration					
		Amount of Erythrocyte		Amount of Leukocyte		Amount of Leukocyte		Amount of Erythrocyte			
	Number of layers	Test 1	Test 2	Test 1	Test 2	Test 1	Test 2	Test 1	Test 2	Leukocyte Decrease (%)	Erythrocyte Decrease (%)
S-1	25 layers of PBT-1	3.55 M/UL	5.99 M/UL	1.93 K/uL	0.5 K/uL	0.0015 K/uL	0.004 K/uL	3.38 M/UL	5.46 M/UL	99.57	6.82
	25 layers of PBT-3										
	50 layers of PA-12										
S-2	50 layers of PBT-3	6.9 M/UL	6.27 M/UL	0.4 K/uL	0.54 K/uL	0.003 K/uL	0.0012 K/uL	6.56 M/UL	5.88 M/UL	99.52	5.80
	25 layers of PBT-2										
	25 layers of PA-12										
S-3	50 layers of PBT-3	6.12 M/UL	7.64 M/UL	0.28 K/uL	0.61 K/uL	0.003 K/uL	0.0027 K/uL	6.1 M/UL	7.04 M/UL	99.24	4.09
	50 layers of PA-12										
S-4	50 layers of PA-12	7.7 M/UL	6.6 M/UL	1.84 K/uL	1.5 K/uL	0.0045 K/uL	0.0082 K/uL	7.2 M/UL	6.3 M/UL	99.61	5.53
	25 layers of PBT-1										
	25 layers of PBT-3										
S-5	50 layers of PA-12	5.47 M/UL	7.25 M/UL	0.96 K/uL	1.0 K/uL	0.009 K/uL	0.0058 K/uL	5.4 M/UL	7.15 M/UL	99.24	1.33
	50 layers of PBT-1										
S-6	30 layers of PBT-1	10.61 M/UL	6.35 M/UL	0.87 K/uL	0.76 K/uL	0.07 K/uL	0.034 K/uL	10.02 M/UL	5.96 M/UL	93.73	5.85
	30 layers of PBT-4										
S-7	30 layers of PBT-1	6.1 M/UL	6.72 M/UL	0.79 K/uL	2.03 K/uL	0.035 K/uL	0.075 K/uL	5.7 M/UL	6.52 M/UL	95.81	4.77
	30 layers of PBT-4										
	20 layers of PBT-3										
S-8	10 layers of PBT-1	6.1 M/UL	6.72 M/UL	1.87 K/uL	2.03 K/uL	0.055 K/uL	0.051 K/uL	5.7 M/UL	6.4 M/UL	97.27	5.66
	20 layers of PBT-2										
	30 layers of PBT-4										
	40 layers of PBT-3										

As seen in Table 4, the filters composed of only PBT polymer (S-6, S-7, and S-8), has achieved much lower leukocyte removal efficiencies than the filters composed of both PBT and PA-12 polymers while both has below 10% erythrocyte loss meeting the standard safety criterion. For example, S-1 filter removed 99.57% of leukocytes (blood leukocyte count from ∼1.93 K/μL to ∼0.002 K/μL) with the erythrocyte loss of 6.82% while, S-8 filter removed 97.27% with the eritrocyte loss of 5.66%.

The results clearly demonstrate the advantages of using N-containing polymer fibers as leukocyte filter material. The S-1–S-5 filters (containing both PBT and PA-12 layers) showed significantly better performance in leukocyte retention than filters containing only PBT fiber layers (S-6–S-8). This is consistent with previous findings that polymer chemistry and surface functionality significantly affect leukocyte reduction: for example, PBT nonwoven fabrics chemically modified with polyvinylpyrrolidone (PVP) achieved ∼96% leukocyte retention with ∼92% RBC recovery in whole blood compared with the untreated PBT.^28^ The adhesion of leukocytes to solid surfaces is a complex process, and the underlying mechanisms are not fully understood. The generally accepted mechanism is that adhesion occurs via van der Waals forces. Factors known to be important for leukocyte adhesion include surface chemistry, surface charge, surface wettability, and surface morphology.^29,30^ According to the literature and analyzed patent data, surfaces carrying hydroxyl groups, carbonyl groups, and amino groups are known to increase leukocyte cell adhesion, while sulfonate groups are known to inhibit adhesion. The effect of carboxylic acid groups is controversial. Like most mammalian cells, leukocytes have a net negative surface charge due to the presence of anionic groups such as phosphate, sialic acid, and carboxylic acid groups in the cell membrane. A positively charged surface of the filter material will attract and retain negatively charged leukocytes. In our case, the basic surface of PBT layers likely provide strong electrostatic adhesion for negatively charged leukocytes, while the PA-12 layers (with nitrogen-containing amides) introduce additional affinity. Surfaces bearing amine groups have been reported to increase leukocyte adhesion (e.g., ∼3× more WBC binding on amine-modified polyurethane versus unmodified).^25^ Thus, the synergistic combination of PBT and PA-12 surfaces appears to enhance leukocyte capture without proportionally trapping more erytrocytes. Although this study provides evidence of the relationship between filtration performance and surface charge (pHpzc), the direct examination of leukocyte-surface interactions using techniques such as zeta potential analysis or microscopic observation of adhered cells in physiological environments is important for elucidating the mechanism. This topic could be addressed in future studies.

While the number of layers and the types of polymers used are critical factors in filtration efficiency, the arrangement and sequence of these layers within the filter have also been investigated. For example, although S-1 and S-4 filters were assembled using the same fiber layers, significant differences in filtration performance were not observed regarding layer arrangements. The S-1 filter achieved 99.57% leukocyte removal efficiency with 6.82% erythrocyte loss, while the S-4 filter where the PA-12 layers are located at the beginning of the flow, has 99.61% leukocyte retention with 5.83% eritrocyte loss.

The effect of the depth filter created using the layers we worked with in the S-1 filter on leukocyte removal was investigated. As shown in Tables 3 and 4, while the layers in the S-1 filter are arranged from small to large pore size, in the S-4 filter, the layer with the largest pore size is placed at the top and the others are placed below. As mentioned earlier, there is no significant difference between the S-1 and S-4 filters in terms of leukocyte removal and erythrocyte loss efficiency. Additionally, there is no noticeable difference in the performance of filters (S-3 and S-5) made with the same number of PA-12 layers and PBT layers with different pore diameters. These findings clearly show that leukocyte retention cannot be attributed solely to size sieving mechanisms. It has been found in previous depth filtration studies that the adsorption mechanisms are more dominant than the mechanical screening mechanisms in fibrous or porous filter systems. In these systems, the retention is mainly dependent on the interaction between the target cells and the inner surfaces of the filter matrix rather than the sieving mechanism. The physicochemical forces such as hydrogen bonds have been found as the major driving force for the adsorption mechanism in porous materials.^31^

Interestingly, while 50 layers of PA-12 are used in the others, 25 layers of PA-12 were used in S-2, yet it demonstrated the same performance as the other PA-12-containing filters. This situation indicates that the number of PA-12 layers has no effect on performance within the limits studied.

International standards for whole-blood leukoreduction vary by jurisdiction but converge on two measurable goals: (1) a target upper limit for residual leukocyte per whole-blood unit and (2) acceptable erythrocyte retention (or conversely, allowable RBC loss) after filtration. The Council of Europe/EDQM technical guidance and numerous European studies apply a stringent target of ≤1.0 × 10^6^ leukocyte per unit for leukoreduced components produced from whole blood, and this threshold is commonly used as the European benchmark.^32,33^ The U.S. regulatory and professional framework (FDA guidance together with AABB practice) allows a somewhat higher limit for whole-blood products that are labeled “leukocytes-reduced”—<5.0 × 10^6^ leucocyte per unit—and additionally specifies a minimum red-cell yield (commonly ≥85% recovery, i.e., ≤15% RBC loss) as an acceptance criterion.^34,35^ Comparing these benchmarks to our results (Table 4): S1–S5 filter designs achieved >99% leukocyte removal, demonstrating robust performance, and preserved red cells with <10% RBC loss (i.e., >90% recovery), which meets or exceeds the FDA/AABB recovery criterion and commonly cited clinical practice goals.

The filtration performance observed in this study, characterized by >99% leukocyte removal and >90% erythrocyte recovery, is highly competitive with established commercial whole blood leukoreduction systems. The Pall RC50 and Sepacell R-500A filters demonstrate mean leukocyte depletion efficiencies of 99.5% and 99.3%, respectively, with RBC recoveries ranging from 87.4% to 92.2%.^36^ Whole blood leukoreduction filters produced by Macoparma, Leucoflex LXT provides performance below the European requirement of <1 × 10^6^ WBC/unit, whilw Leucoflex MTL1 provides reliable performance below the AABB requirement of <5 × 10^6^ WBC/unit.^37^ The multilayer PBT/PA-12 filters developed in this study achieved >99% leukocyte removal with <10% erythrocyte loss (>90% erythrocyte recovery), indicating that their performance is within the range reported for clinically used leukoreduction systems such as AABB practice. However, it should be noted that the present work evaluates laboratory-scale prototype filters in a custom experimental setup. Further studies addressing large-scale melt-blown production, control of fiber and layer uniformity, integration into medical-grade filter housings, and comprehensive biocompatibility validation will be necessary before clinical translation can be considered.

These results demonstrate that optimizing the filter design, particularly through adjustments to the number of layers, structure, and material properties, can achieve both efficient leukocyte removal and minimal erythrocyte loss, thus meeting strict international leukocyte reduction standards.

Overall, the results highlight that, in addition to polymer selection and pore size, the sequence and distribution of filter layers are key parameters that can significantly impact both leukocyte removal efficiency and erythrocyte retention. Optimizing these structural parameters could enable the production of improved filters tailored to specific clinical needs.

## 4 Conclusion

This study successfully demonstrated the development of melt-blown nonwoven filter media using PBT and PA-12 polymers for the effective removal of leukocytes from whole blood. Multilayer filters combining PBT and PA-12 fibers attained the highest performance: they consistently removed >99% of leukocytes with <10% erythrocyte loss, whereas PBT-only filters were less effective. The superior performance of mixed-material filters is attributed to synergistic surface chemistry of N-containing polymer PA-12 and PBT’s mildly basic character enhancing leukocyte adhesion. Notably, varying the arrangement of the layers with varying pore sizes in the range of roughly 15–68 μm, had little effect on leukocyte retention efficiency, implying that leukocyte retention was dominated by adhesion interactions rather than simple sieving by pore size.

However, while some filter designs could be adapted to meet the requirements of leukoreduction as specified in certain countries, none of them could meet the most demanding criterion of <10^6^ leukocytes per unit. The results of this study, based on the mechanistic insights, suggest that the process of leukocyte removal is more dependent on adsorption-based interactions than simple sieving; hence, future studies should aim at adopting multidimensional approaches that aim to optimize fiber morphology, surface chemistry, and hierarchical structure in a more integrated manner, rather than merely focusing on the reduction of pore size. In this regard, some of the most promising avenues include the optimization of the PBT/PA-12 blend ratio during melt-blown fabrication to produce smaller fibers and more heterogeneous and high surface area structures, modifying the PBT or PA-12 surfaces through grafting (e.g., quaternization reactions or the introduction of functional groups to increase leukocyte adhesion), developing biomimetic gradient pore structures that integrate adsorption with controlled permeability and limited sieving effects, and assessing the impact of partial substitution with biobased or biodegradable polymers.

This study clearly demonstrates the structure–property-performance relationships in PBT/PA-12 melt-blown filters, and the integrated optimization strategy outlined above provides a viable route to meet the leukoreduction requirements for transfusion purposes.
